# Seasonal Influenza in Children With Cancer

**DOI:** 10.7759/cureus.72785

**Published:** 2024-10-31

**Authors:** Preston Daniels, Lara Danziger-Isakov, William Otto

**Affiliations:** 1 Internal Medicine, Rush University Medical Center, Chicago, USA; 2 Pediatric Infectious Diseases, Cincinnati Children's Hospital Medical Center, Cincinnati, USA

**Keywords:** antiviral, cancer, children, influenza, vaccine

## Abstract

Influenza is a major cause of morbidity and mortality for pediatric cancer patients. We review important aspects in the management of influenza, including virology, epidemiology, clinical presentation, diagnostic testing, and antiviral treatment. Topics that are addressed include optimal treatment of influenza in children with cancer as well as strategies for prevention. This includes both pre-/post-exposure chemoprophylaxis and vaccination.

## Introduction and background

Influenza viruses are RNA viruses that infect both humans and a variety of animal species. Annual influenza outbreaks are a major cause of morbidity and mortality for children [1]. Children with cancer are at increased risk of influenza-related hospitalization and death compared to immunocompetent children [2,3]. Chemotherapy impairs normal immune function, resulting in lower response to influenza vaccination, decreased ability to control infection, and increased risk of prolonged viral replication. Children with cancer have increased rates of complications of influenza infection. Influenza infection may also cause interruptions in a child’s chemotherapy, potentially leading to suboptimal treatment [2,4]. Effective prevention, diagnosis, and treatment are paramount to reduce complications of influenza. As the clinical presentation of influenza is similar to that of other respiratory viruses, delays in diagnosis may prevent timely initiation of therapy in this high-risk population. In this article, we review important aspects in the management of influenza in children with cancer, including epidemiology, clinical presentation, diagnosis, treatment, and prevention.

## Review

Virology

Influenza viruses are orthomyxoviruses composed of eight single-stranded RNA segments, viral proteins, and viral polymerases within a lipid bilayer [5]. There are four influenza subtypes: A, B, C, and D. Influenza A and B cause most human infections [1]. Influenza viruses possess two glycoproteins that mediate infection: hemagglutinin (HA) and neuraminidase (NA). HA binds to sialic acid on the cell surface. This allows the virus to undergo endocytosis into the cellular endosome. The matrix protein M2, an ion channel, then acidifies the viral particle. This fuses the virus with the endosome and releases viral RNA and proteins into the cell. The virus then replicates and assembles daughter virions. After mature virions assemble on the cell surface, NA cleaves the bond between cellular sialic acid and the viral HA, releasing the budding virion.

**Figure 1:**
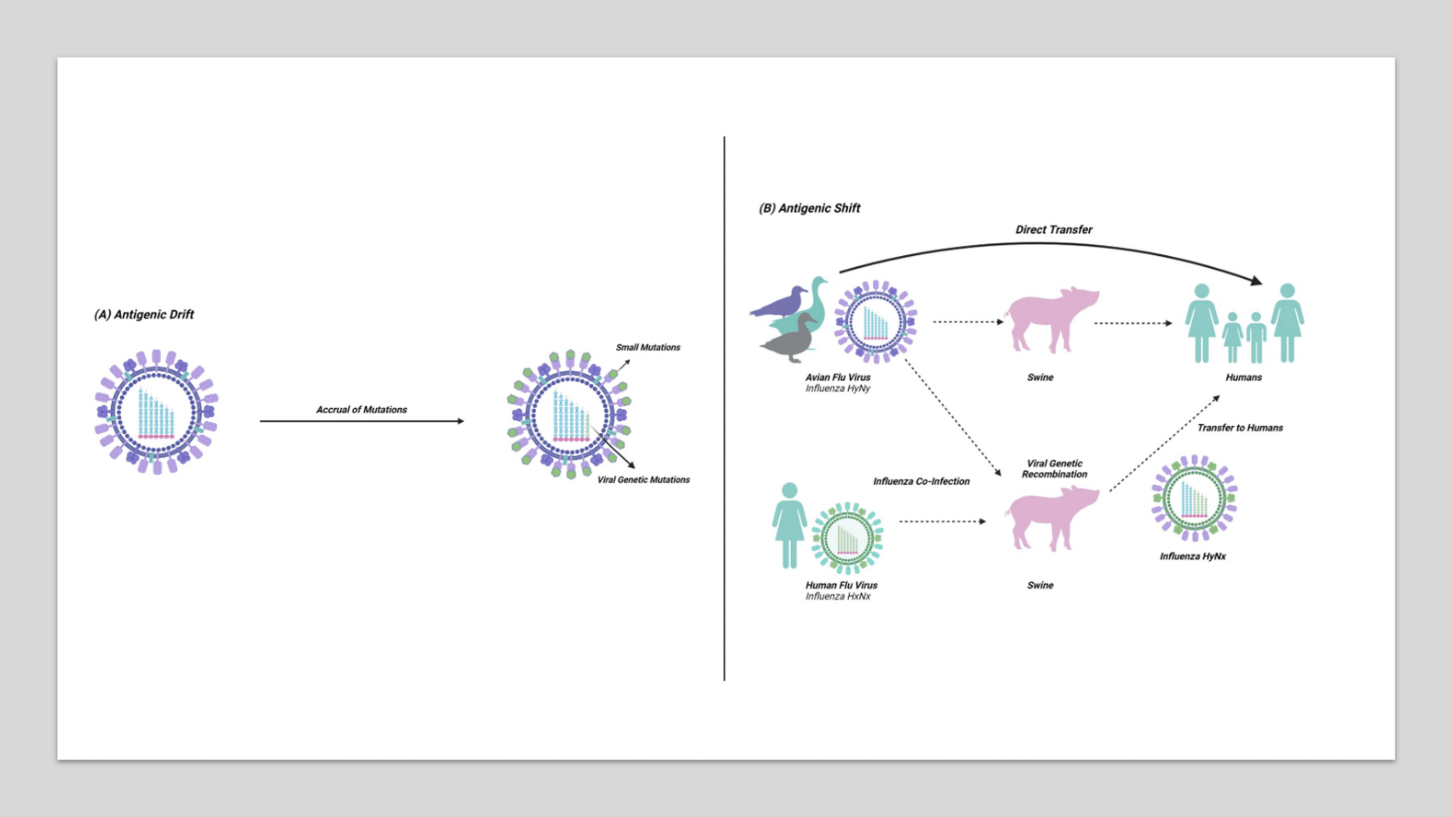
In antigenic drift (A), small mutations lead to alterations in the expressed HA or NA glycoproteins, allowing for immune evasion. In antigenic shift (B), co-infection with multiple strains allows for genetic recombination, leading to novel influenza strains with the potential to cause pandemics. HA: hemagglutinin; NA: neuraminidase. Figure created with BioRender.com.

Influenza strains are characterized by their HA and NA. For example, the H1N1 subtype of influenza A expresses the H1 and N1 subtypes of each glycoprotein, respectively. As the primary influenza antigens, HA and NA are the targets of neutralizing antibodies and experience intense selective pressure. This leads to mutations that alter glycoprotein conformation to allow viruses to escape key antibodies [1,6]. This phenomenon, known as antigenic drift, continually changes viral antigenicity and is a major driver of annual influenza outbreaks (Figure 1A). Influenza also experiences drastic changes in antigenicity due to recombination events that occur during co-infections with multiple strains [1]. Large amounts of genetic material are transferred between strains, generating novel influenza strains (Figure 1B). Known as antigenic shifts, these events create strains distinct from common circulating strains, leading to influenza pandemics [5].

Epidemiology and risk factors

Nearly 10% of children develop symptomatic influenza each year [7]. The prevalence of influenza infection in children with cancer mirrors that of the community [8]. Influenza was reported as the most common viral infection in a cohort of children with acute lymphoblastic leukemia [9], although other respiratory viruses occurred more frequently in other cohorts [10-13]. Many studies describing influenza in children with cancer have been small, single-center studies or focused on the 2009 H1N1 pandemic [2,3,14-16].

One retrospective study utilized administrative data to evaluate the burden of influenza hospitalizations in acute lymphoblastic leukemia. The attributable mortality of influenza infection was 1.0%, and mortality was highest in children <2 years of age and those in the first six months after leukemia diagnosis [4]. Similar studies have not been performed for other pediatric malignancies, so the epidemiology of influenza in those populations is not well described.

A number of studies have identified risk factors for severe influenza infection in children with cancer. Lymphopenia has been identified as a risk factor for hospitalization [2], though not in every cohort [3,17]. Receipt of corticosteroids and intensive chemotherapy regimens have been linked to severe infection [18]. Older age was identified as a risk factor for influenza hospitalization in one cohort [3], though another study associated younger age with increased mortality [4]. Delay in antiviral therapy or bacterial superinfection has been linked to mortality [18].

Transmission and clinical illness

Influenza epidemics occur annually, with the typical influenza season in the Northern Hemisphere lasting from October to May [1]. The number of infections typically peaks from December to February. The number of circulating influenza strains changes each year, but the presence of two to three strains in a community has been associated with a longer influenza season and multiple peaks in influenza infections [6].

Transmission

Infection is spread primarily via respiratory droplets generated by coughing or sneezing. Transmission requires close contact between individuals, as there is limited evidence to support long-distance transmission [1,7]. Infection can be transmitted via contact with contaminated surfaces, such as an unwashed hand. Virus particles can remain infectious on hard surfaces for up to 24 hours [19].

Infected patients can transmit the virus in the 24 hours prior to symptom onset. In immunocompetent patients, shedding of virus in nasal secretions is highest during the first few days of illness and ends after approximately seven days. In immunocompromised patients, viral shedding occurs at higher levels and for longer periods, with viral particles detected for weeks after infection [20]. Patients receiving high-dose steroids can be expected to experience extended periods of viral shedding [21].

Clinical Manifestations

Clinical manifestations of influenza in children with cancer are similar to immunocompetent children. After exposure, children generally develop symptoms within one to four days. Fever is the hallmark of influenza infection, and high, spiking fevers are common. In pediatric cancer patients, fever may last for one to four days, with symptoms persisting for six to 18 days [6,7]. The onset of illness is sudden, and fever is often accompanied by malaise, myalgias, headache, and chills or rigors. Respiratory symptoms, such as congestion, cough, and sore throat, are prominent. Immunocompromised children may present without fever, and cough may be less prominent in younger children [1]. Gastrointestinal symptoms, such as abdominal pain, diarrhea, or nausea/vomiting, are uncommon in pediatric influenza.

Influenza infection in pediatric patients ranges in severity from mild infection to acute respiratory failure and septic shock. Children with influenza can present with lower respiratory tract infection such as bronchiolitis or pneumonia [6]. A feared complication of influenza infection is bacterial superinfection, most commonly with *Streptococcus pneumoniae* or *Staphylococcus aureus* [1,6]. Secondary bacterial pneumonia is associated with more severe clinical outcomes, including septic shock, acute respiratory distress syndrome, and multiorgan failure.

Non-respiratory Complications of Infection

Influenza infection is associated with a variety of non-respiratory clinical syndromes, which occur in up to 30% of patients [3]. Neurologic complications occur most commonly in young children and can include febrile seizures, exacerbation of a pre-existing seizure disorder, or encephalopathy [1]. A more fulminant syndrome known as acute necrotizing encephalopathy presents with coma and parenchymal necrosis on neuroimaging [6]. Influenza infection can also present with calf tenderness and refusal to walk due to acute myositis. Patients can have mild pain with elevated serum creatine kinase levels, which can progress to rhabdomyolysis [1]. Patients may have a mild elevation in liver transminases. A more severe hepatopathy can occur in children given aspirin during influenza infection. Known as Reye syndrome, patients can progress to hepatic failure, encephalopathy, and death [1]. For this reason, children with influenza should not be given aspirin. Cardiac complications from influenza, such as myocarditis or pericarditis, are rare.

Diagnosis

Diagnostic testing for influenza should be pursued promptly for children with cancer presenting with an influenza-like illness, as a positive test result would be an indication to initiate antiviral treatment. There are several different types of assays for the diagnosis of influenza. The sensitivity and specificity for a diagnostic test vary not only by the specific method utilized but also by the time from illness onset, source of the specimen, specimen quality, and specimen processing.

Molecular tests detect influenza virus nucleic acid or RNA. Clinically available molecular tests include rapid molecular assays, reverse-transcription polymerase chain reaction (RT-PCR) tests, and other nucleic acid amplification tests (NAATs). Molecular tests have the best performance characteristics of available diagnostic assays, with high sensitivity (90-95%) and specificity (>99%) [22]. Additionally, molecular tests can differentiate between influenza A and B subtypes and identify other respiratory viruses [23]. Rapid molecular tests provide results in approximately 15-40 minutes, while other assays require several hours to be completed.

Rapid influenza diagnostic tests (RIDTs) and immunofluorescence assays detect influenza antigens. The majority of these tests are RIDTs, immunoassays that can detect influenza A or B antigens in 10-15 minutes. RIDTs can identify influenza A or influenza B but not specific viral subtypes [24]. While RIDTs have high specificity (>90%), they only have low-to-moderate sensitivity (50-70%) [25]. Due to their low sensitivity, false negative results are common during peak influenza seasons, and a negative RIDT should be confirmed with molecular testing in children with cancer. Immunofluorescence assays (IFAs) utilize immunofluorescent staining to detect influenza A and B antigens but, similar to RIDTs, cannot identify specific viral subtypes. Most IFAs take one to four hours to perform, though there is a rapid IFA that can be completed in approximately 15 minutes. Immunofluorescence assays also have moderate sensitivity and high specificity.

Molecular tests are the test-of-choice for the diagnosis of influenza in children with cancer. The Infectious Diseases Society of America (IDSA) recommends the use of rapid molecular assays for outpatients with suspected influenza infection [26]. RT-PCR testing or other molecular tests should be utilized for hospitalized patients, and the IDSA specifically recommends the use of multiplex PCR assays for hospitalized immunocompromised patients.

Treatment

**Figure 2:**
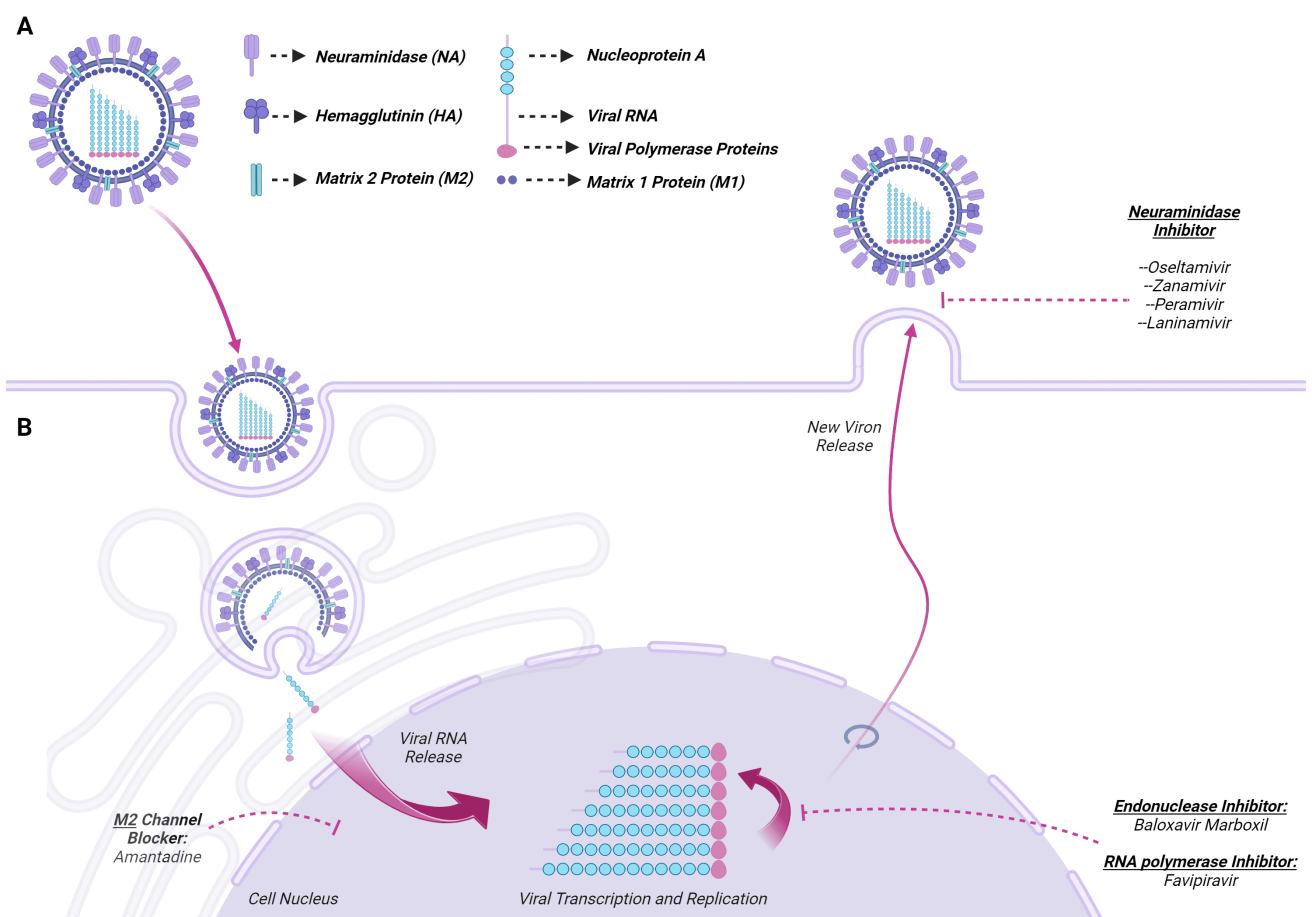
Antivirals active against influenza. Figure created with BioRender.com.

Antiviral therapy is the mainstay of influenza treatment for children with cancer. As children with cancer have an increased risk of severe influenza infection and subsequent complications, all patients with documented influenza infection should receive treatment. However, in a recent study of children hospitalized with influenza, only 87% of children with cancer received antiviral treatment [27]. The greatest clinical benefit is achieved when antiviral treatment is started within 48 hours of symptom onset whenever possible [26]. If there is a delay in obtaining the results of influenza diagnostic testing, then empirical therapy should be initiated. A summary of antiviral drugs used to treat influenza is shown in Figure 2 and Table 1 [6].

**Table 1:** Antiviral drugs for influenza.

Medication	Treatment use	Chemoprophylaxis use	Comments
Oral oseltamivir	Five days If younger than one year old: 3 mg/kg/dose twice daily; if one year or older, dose varies by child’s weight: 15 kg or less, the dose is 30 mg twice a day; >15 to 23 kg, the dose is 45 mg twice a day; >23 to 40 kg, the dose is 60 mg twice a day; >40 kg, the dose is 75 mg twice a day	Seven days Not recommended unless critical if child is younger than three months old. If child is three months or older and younger than one year old: 3 mg/kg/dose once daily. If one year or older, dose varies by child’s weight: 15 kg or less, the dose is 30 mg once a day; >15 to 23 kg, the dose is 45 mg once a day; >23 to 40 kg, the dose is 60 mg once a day; >40 kg, the dose is 75 mg once a day	Giving with food may reduce gastrointestinal discomfort
Inhaled zanamivir	10 mg (two 5 mg inhalations) twice daily for seven days	10 mg (two 5 mg inhalations) once daily for five days	Not recommended for use in children with underlying respiratory disease, including those with asthma and cystic fibrosis, because of the risk of severe bronchospasm; also not recommended for severe influenza, including those requiring hospitalization
Intravenous peramivir	(Six months to 12 years of age) One 12 mg/kg dose, up to 600 mg maximum, via intravenous infusion for a minimum of 15 minutes	Not recommended	Consider for patients with difficulty tolerating oral/inhaled forms
Oral baloxavir marboxil	For children five years and older: ≤20 kg, single dose of 2 mg/kg; 20 kg to <80 kg, single dose of 40 mg; ≥80 kg, single dose of 80 mg	FDA-approved for post-exposure prophylaxis for persons aged five years and older. Dosage is the same as for treatment.	Newly approved; different mechanism. Interactions with polyvalent cation-containing products (calcium, iron, magnesium). Avoid co-administration with antacids, dairy products, or cation supplements

Antiviral Medications for Treatment of Influenza

Neuraminidase inhibitors are the primary anti-influenza drugs available for treatment of influenza for children with cancer [7,26]. These drugs are active against both influenza A and B strains and interrupt the spread of infection by blocking the release of progeny virions from infected cells.

Oseltamivir is the most frequently used NA inhibitor and is approved for use in children >14 days of age. Oseltamivir is recommended for treating hospitalized patients with influenza. In a meta-analysis of clinical trials that included 2,561 children with influenza, oseltamivir was shown to reduce the duration of illness by approximately 18 hours [28]. The impact was increased when oseltamivir was administered within 24 hours of symptom onset, a finding confirmed by a retrospective study with a cohort of 55,799 children hospitalized with influenza [29]. Early administration of oseltamivir was associated with shorter hospital stay and lower odds of complications of infection.

Both zanamivir and peramivir are recommended for uncomplicated influenza infection. Zanamivir is an inhaled NA inhibitor that is as effective as oseltamivir in reducing fever in children with influenza [30]. Peramivir is an intravenous antiviral drug used to treat infants or children with influenza who unable to tolerate oral therapy and has similar effectiveness to oseltamivir [31]. Another NA inhibitor, laninamivir, is approved for treatment of influenza in Japan and is administered via inhalation.

Baloxavir marboxil is a cap-dependent endonuclease active against both influenza A and B strains and is approved for treatment of uncomplicated influenza. Baloxavir has been shown to reduce influenza symptoms in healthy adults and children with uncomplicated influenza [32-34]. However, the development of escape mutants with reduced susceptibility to baloxavir occurred in 10% of patients after a single dose of therapy [34]. Baloxavir is not recommended for treatment of severely immunocompromised individuals due to a lack of data supporting its use and the potential for antiviral resistance.

Other antivirals include the adamantane drugs and polymerase inhibitors. High levels of adamantane resistance in circulating influenza A strains have rendered these drugs ineffective [35], and their use is no longer recommended. Favipiravir, an influenza RNA polymerase inhibitor, is used in Japan for the treatment of influenza.

Combination therapy using baloxavir and NA inhibitors is one treatment option for immunocompromised patients. In one study, patients were randomized to receive either baloxavir plus an NA inhibitor or a placebo plus the NA inhibitor [36]. Combination therapy did not show benefit in reducing time to clinical improvement but did shorten duration of viral shedding and decreased emergence of antiviral resistance in the combination therapy arm. There have been no studies conducted solely using pediatric patients, and combination therapy is not routinely utilized.

Adjunctive Therapies

While immunomodulatory therapies play an important role in the treatment of acute respiratory distress syndrome due to COVID-19 [37], their role in severe influenza is less clear [26]. A meta-analysis of observational studies reported that corticosteroid administration was associated with increased mortality and hospital-acquired infection [38]. Data from randomized clinical trials of adjunctive corticosteroid treatment in influenza patients have not been published [26]. Similarly, data for other immunomodulatory agents are limited to case series or other observational studies. A small randomized controlled trial of convalescent plasma performed during the H1N1 pandemic reported that treatment with hyperimmune immunoglobulin was associated with a lower viral load and reduced mortality [39], but high-titer convalescent plasma conferred no clinical benefit in a subsequent multicenter randomized controlled trial [40]. Immunomodulatory agents are not recommended as part of routine care.

Prevention of influenza infection

There are multiple avenues to prevent influenza transmission and the incidence of infection, ranging from public health policies to vaccination [41]. These interventions contribute to a reduction in influenza infections but do not eliminate influenza transmission. A combination of these actions should be effective in reducing the risk of influenza infection for children with cancer.

Non-pharmaceutical Interventions

Personal hygiene actions such as handwashing and respiratory hygiene are effective at reducing influenza infections. Children with cancer, as well as household contacts, should be counseled to cover their mouth and nose when sneezing or coughing, wash their hands after sneezing or coughing, and maintain physical distancing from ill-appearing individuals. Hand hygiene can be performed either with soap and water or with alcohol-based hand sanitizer.

Some have advocated for the inclusion of mask-wearing as a potential intervention to reduce influenza infection, as wearing masks reduces respiratory illnesses in both community and healthcare settings [42]. Other analyses did not identify a reduction in respiratory viral infections with use of masks [43]. It is not unreasonable to recommend that children with cancer wear masks during periods of increased influenza activity, but it should be remembered that they will likely need to wear them any time they are in a public place or around household members.

Infection prevention and control precautions can decrease transmission of influenza in healthcare settings [6]. Patients with suspected respiratory viral infection should be given masks to wear and placed in a private room. Healthcare providers should follow droplet precautions, which include a mask and eye protection. If a patient requires transfer out of the isolation room, they should be masked. All patients with suspected or confirmed influenza should be taking droplet precautions while in a healthcare facility for a total of seven days after the onset of symptoms or until 24 hours after the resolution of symptoms, whichever is longer.

Vaccination

Influenza vaccine is the most effective method to reduce both influenza infection and complications of infection. Influenza vaccines are updated each year to protect against the influenza A and B strains considered most likely to cause illness during the upcoming influenza season. Vaccination does not prevent all cases of influenza but offers outsize benefit for children, a population in which the majority of hospitalizations and deaths occur in unvaccinated individuals [7]. All children over the age of six months are recommended to undergo influenza vaccination each year [7,44]. Children under the age of nine years who are being vaccinated for the first time are recommended to receive two doses of the influenza vaccine at least four weeks apart. Any age-appropriate inactivated influenza vaccine is suitable; one product is not preferred over another [7]. Live-attenuated influenza vaccines should not be given to children with cancer due to the potential risk of vaccine-related infection [44].

The immune response to vaccination is impacted by the duration, schedule, and modalities of chemotherapy [45,46]. Children receiving intensive chemotherapy (such as induction chemotherapy for acute leukemia) or those who have received anti-B-cell monoclonal antibodies in the previous six months are not expected to mount an effective response to influenza vaccination. Vaccination should be deferred for those patients until they will be more likely to respond appropriately. Other children with cancer should receive the influenza vaccine annually. Administration of the influenza vaccine should occur at least two weeks prior to administration of chemotherapy to allow for the development of an immune response [47].

It is well established that children with cancer mount a lesser response to vaccination when compared to healthy children. A number of studies have evaluated the immunogenicity of influenza vaccines in children with cancer, which were nicely summarized by Furlong and Kotecha [46]. Immunogenicity varied widely depending on the study population and phase of chemotherapy. Seroprotection was achieved in 35-96% of patients for the H1N1 strain, 25-98% for H3N2, and 15-100% for the influenza B strain included in the vaccine. Several factors were consistently associated with improved immune response to influenza vaccination, including lymphocyte count of >1.0 x 10^3^ cells/microliter and normal or high immune globulin levels. Children with solid tumors were more likely to have improved immune response [48], consistent with the lower intensity and shorter duration of chemotherapy for solid tumors.

Several strategies have been proposed to increase the immune response to vaccination. A two-dose vaccine schedule in a single season is one such strategy, based on an Australian study that reported that vaccine-naïve children <10 years of age with cancer who received two doses of trivalent influenza vaccine were more likely to develop an immune response than previously vaccinated children who received one vaccine dose [48]. Another strategy is the use of high-dose inactivated influenza vaccines, which contain four times the dose of the standard-dose vaccine. A high-dose vaccine has been shown to be more immunogenic than a standard-dose influenza vaccine in pediatric cancer patients without a significant increase in adverse events [49]. However, there is limited data to support strategies to boost vaccine immunogenicity for children with cancer, and there is no recommendation for their use. Lastly, vaccination of the family members of children with cancer, as well as all hospital staff caring for those children, is strongly recommended.

Chemoprophylaxis

Chemoprophylaxis should not replace vaccination but may play a valuable role in preventing symptomatic influenza in children with cancer. Chemoprophylaxis can be utilized in two ways: prior to exposure to influenza or after a child has been exposed to a contact with confirmed influenza.

Pre-exposure prophylaxis is rarely used but can be considered for unvaccinated children or significantly immunocompromised children for whom vaccination is not expected to be effective. Some patients may only require a short duration of chemoprophylaxis until they can undergo vaccination. Others, such as children who have recently undergone hematopoietic cell transplantation, may require prophylaxis for the entire influenza season. It is recommended that NA inhibitors be utilized for chemoprophylaxis [26]. In a randomized trial composed largely of adult solid organ transplant recipients, the efficacy of oseltamivir prophylaxis to prevent influenza diagnosed by PCR was 79.9% [50]. It should be noted that the relatively high efficacy of chemoprophylaxis correlates to a modest absolute risk reduction of infection, so the number needed to treat to prevent influenza infection is high.

Post-exposure chemoprophylaxis should be considered for high-risk children for whom vaccination is expected to be less effective after household exposure to influenza. Once-daily antiviral prophylaxis with oseltamivir or zanamivir, given for seven days after the last exposure, has been shown to effectively decrease the likelihood of symptomatic influenza [51,52]. As with pre-exposure prophylaxis, there are limited data in children [53]. In an open-label trial of post-exposure prophylaxis, receipt of oseltamivir post-exposure prophylaxis reduced the likelihood of febrile influenza by 64% in children (95% CI 15.8-85.0%) [54]. Once-daily prophylaxis with a neuraminidase inhibitor should be started as soon as possible after exposure. If >48 hours have elapsed since exposure, patients should be monitored closely, with full-dose therapy started when symptoms develop. Some experts recommend giving twice-daily dosing to reduce the risk of subtherapeutic dosing, especially if >48 hours have elapsed since exposure and empirical therapy may be needed. There are no data to support this strategy.

Future directions

Influenza viruses are significant pathogens for children with cancer, and much remains to be learned about the impact of influenza infections in that high-risk population. Further research is needed to understand the incidence of influenza in children with cancer other than leukemia, as well as how influenza impacts the chemotherapy schedule. Research is needed to identify the optimal treatment strategy for hospitalized children with severe influenza. Vaccination remains the cornerstone of influenza prevention, but strategies to optimize the immune response to influenza vaccination and develop more immunogenic vaccines are needed.

## Conclusions

Influenza causes significant clinical disease in children with cancer and can be associated with high morbidity and mortality. The epidemiology of influenza in pediatric cancer patients largely mimics that of immunocompetent children, although children with cancer are at high risk for severe infection and the development of complications of infection. Diagnosis in this population relies on the use of molecular diagnostic testing. Oseltamivir is the backbone of treatment for influenza infection, and children with cancer should have treatment initiated promptly when the diagnosis is made. Vaccination is the main route of prevention of influenza infection, but chemoprophylaxis can be considered for patients that will not respond to immunization. Continued efforts are needed to improve management of this infection in this vulnerable population.
